# A population-based study of immunohistochemical detection of p53 alteration in bladder cancer

**DOI:** 10.1038/sj.bjc.6601748

**Published:** 2004-04-06

**Authors:** K T Kelsey, T Hirao, A Schned, S Hirao, T Devi-Ashok, H H Nelson, A Andrew, M R Karagas

**Affiliations:** 1 Department of Cancer Cell Biology, Harvard School of Public Health, 665 Huntington Avenue, Boston, MA 02115, USA; 2 Department of Pathology, Dartmouth Medical School, Hanover, NH 03756, USA; 3 Department of Environmental Health, Harvard School of Public Health, 665 Huntington Avenue, Boston, MA 02115, USA; 4 Department of Family and Community Medicine, Dartmouth Medical School, Hanover, NH 03756, USA

**Keywords:** bladder cancer, P53, immunohistochemistry, mutation, population-based

## Abstract

The molecular pathology of bladder cancer has been the subject of considerable interest and mutation of the p53 gene, which has been associated with more invasive bladder cancer, has been widely studied. Further, there is evidence that p53 inactivation (either mutation or protein dysregulation), independent of stage, may be predictive of bladder cancer progression. In an effort to avoid possible biases associated with selection of more advanced cases, we examined p53 inactivation in a population-based study of bladder cancer in New Hampshire, using both mutation and immunohistochemical methods. We found the overall prevalence of mutation to be approximately 10%, while immunohistochemical analysis suggests that approximately 66% of the tumours have dysregulated p53 at the protein level. There was a significant association of mutation with persistent p53 staining, but there remained a marked number of tumours discordant for mutation and aberrant p53 immunohistochemistry. Based upon immunohistochemical staining alone, intensity rather than extent of p53 staining was more strongly related to tumour invasiveness. Additionally, all tumours with a mutation in exon 8 stained intensely. Taken together, this suggests that intense staining represents a distinct phenotype of dysfunctional protein. Our data indicate that population-based approaches to somatic alteration of p53 in bladder cancer are crucial to understanding the relationship of p53 changes to aetiology and the outcome of this disease, and further suggest that the pattern of immunohistochemical staining may represent distinct, discernible phenotypes.

Approximately 60 000 new cases of bladder cancer will be diagnosed in the United States in 2003 (Jemal *et al*, 2003). This disease occurs predominantly in Caucasians, with other racial/ethnic groups having at least a 50% lower incidence. Regardless of racial or ethnic group, the male-to-female ratio of disease incidence approaches three to one (Jemal *et al*, 2003). Bladder cancer occurs overwhelmingly in one histologic cell type (transitional cell), although squamous cell carcinoma and adenocarcinoma of the bladder do occur rarely (Silverman *et al*, 1996). At the time of its initial diagnosis, bladder cancers tend to be of lower stage (Larsson *et al*, 2003). These noninvasive tumours tend to have a significantly higher survival rate (Larsson *et al*, 2003; Soloway *et al*, 2003). Yet, many early-stage bladder cancers will recur and remain superficial and noninvasive for extended periods, while a minority can become invasive and life threatening. The factors that are responsible for altering the noninvasive phenotype of these cancers remain poorly appreciated.

It is clear that inactivation of the p53 pathway is important in bladder cancer, as the number of tumours having p53 mutations increases with the degree of invasiveness of the tumour. At the same time, a large fraction of early-stage disease displays altered p53 staining, suggesting that the p53 pathway is disrupted in some fashion. The significance of this discordance is unclear; stabilisation of nuclear protein could indicate that the protein is mutant, but might also reflect an altered activation pathway for p53 or disrupted turnover kinetics. P53 protein in the cell is tightly regulated, with negligible antibody staining noted in normal tissues. However, inhibition of degradation, mutation or enhanced production of p53 protein (or some combination of these) could all result in antibody recognition of the protein and thus abnormal staining.

Our understanding of the p53 pathway in bladder cancer derives largely from hospital-based and retrospective studies that have captured advanced stage patients undergoing surgery for their disease (Spruck *et al*, 1994; Silverman *et al*, 1996; Smith *et al*, 2003; Soloway *et al*, 2003). Population-based approaches have been used to avoid possible biases attributable to the study of aggressive and invasive disease (Berggren *et al*, 2001; Larsson *et al*, 2003). Overall, the prevalence of mutation has varied quite widely, with the larger and population-based studies tending to report a smaller prevalence of mutation (Sidransky *et al*, 1991; Fujimoto *et al*, 1992; Cordon-Cardo *et al*, 1994; Berggren *et al*, 2001). Studies indicate that the presence of mutation at p53 markedly increases with stage and grade of disease (Spruck *et al*, 1994; Berggren *et al*, 2001; Smith *et al*, 2003). This has led some to suggest that there could be a very sharp boundary between the different disease categories (Larsson *et al*, 2003). The few studies that compared both immunohistochemical staining and p53 mutation found appreciable staining in tumours harbouring mutations, as expected (Esrig *et al*, 1993; Cordon-Cardo *et al*, 1994; Oyasu *et al*, 1995; Vet *et al*, 1995; Abdel-Fattah *et al*, 1998; Bernardini *et al*, 1999). These studies also observed a surprising number of discordant samples where p53 immunohistochemistry (IHC) was positive but no mutation was detectable.

Both the early-stage tumours lacking persistent staining and those cancers with p53 staining may later undergo p53 mutation and become aggressive and invasive. Thus, one might expect the immunohistochemical p53 staining pattern to differ between tumours with stabilised wild-type p53 protein and those in which the protein is mutant. To examine the characteristics of positive staining for p53 protein in bladder tumours systematically, we evaluated both p53 mutation and immunohistochemical staining of p53 in a population-based sample of bladder cancers from inhabitants of New Hampshire. We sought to determine the associations of p53 mutation with clinical stage and grade of tumour in the general population setting and elucidate the relationship of staining characteristics, mutation and the clinical and pathologic features of the tumours.

## MATERIALS AND METHODS

### Study population

Residents of New Hampshire aged 25–74 years, diagnostic from July 1, 1994 to June 30, 1998, were identified by a rapid reporting system of the New Hampshire State Cancer Registry (Karagas *et al*, 1998). Briefly, by state law, practitioners are required to provide a report of cancer patient after diagnosis. Study participants completed an extensive interview to obtain information on demographic traits and carcinogen exposure (Karagas *et al*, 1998).

Pathology reports and paraffin-embedded tumour specimens were requested from the treating physician/pathology laboratories. Bladder tumours were reviewed by one pathologist and classified according to the WHO classification of bladder tumours. DNA was extracted by a previously reported method (Nelson *et al*, 1998). Briefly, three 20-*μ*m sections were cut and transferred into tubes with digestion buffer. After microwave treatment and centrifugation, the paraffin ring was removed. Paraffin-free tissue pellets were suspended in digestion buffer with proteinase K. Supernatants containing DNA lysate were boiled to denature the residual protease.

### Immunohistochemistry

Immunohistochemical staining of paraffin-embedded slides was performed using the avidin–biotin complex technique. For each case, a single representative slide was selected for staining and histologic evaluation. Briefly, slides were deparaffinised and hydrated into water. Slides underwent antigen retrieval in Citra solution using the Biocare Decloaking Chamber (Biocare Medical, Walnut Creek, CA, USA). Staining of p53 was performed using a monoclonal antibody (BioGenex, San Ramon, CA, USA) at a 1 : 100 dilution on the Optimax I-6000 Immunostainer (BioGenex). An appropriate positive control was used in each staining run, and each slide was stained with a negative control. The intensity of nuclear staining was graded on a semiquantitative scale (0–3), rating intensity in the dominant pattern within the tumour. In addition, the percentage of positively staining tumour cells was scored (negative, 1–9%, 10–49%, or ⩾50%).

### Mutation analysis

SSCP analysis of p53 exons 5–9 was performed on all bladder tumour samples. Exons were amplified by PCR containing fluorescence dye-labelled primers. Previously reported primer sequences for each exon were used (Toguchida *et al*, 1992). In all, 1 *μ*l of PCR product and 1 *μ*l TAMRA-350 size standard (Applied Biosystems, Foster City, CA, USA) were denatured in 4 *μ*l of formamide/blue-dextran denaturing buffer at 95°C for 5 min, and then loaded onto MDE gels. Gel electrophoresis was carried out on a DNA autosequencer ABI Prism 377 (Applied Biosystems), with an external cooling system (Thermo NESLAB Portsmouth, NH, USA) attached for gels run at 25°C. Genescan 3.1 software (Applied Biosystems) was used for fragment analysis. Samples with variant SSCP bands were purified using Centri-Sep columns (Princeton Separations Adelphia, NJ, USA) and directly sequenced by a DNA autosequencer ABI Prism 377 using the Big Dye Terminator v3.0 sequencing kit (Applied Biosystems) according to the manufacturer's instructions. The data were analysed with the Sequencing Analysis 3.3 software (Applied Biosystems) and Sequencher 4.1 software (Gene Codes Corporation, Ann Arbor, MI, USA).

### Statistical analysis

We examined the prevalence of p53 mutation, persistent staining (⩾50%) and strong intensity (3+) by patient demographic characteristics and tumour histology and stage. We also used the disease group classification described by Larsson (Larsson *et al*, 2003), which distinguished patient survival. We estimated the prevalence odds ratios (POR) and 95% confidence intervals (CI) of patient demographic and tumour traits for each p53 mutation, persistent staining and strong intensity separately, using a log-linear model with adjustment for the other factors. We also calculated the sensitivity, specificity and predictive positive values to evaluate the concordance between p53 mutation and the two immunohistochemistry parameters (i.e., persistent staining and strong intensity). We further performed a logistic regression analysis to contrast those with tumours containing both mutation and positive immunohistochemistry with tumours that had no evidence of either a p53 mutation or positive immunohistochemistry. Lastly, we compared the number of tumours with intensity staining and the number of tumours with invasive disease by the type of p53 mutation using a *χ*^2^ test. Due to the sparse numbers of tumours, we grouped the low-grade tumours (G1 and G2) together when necessary.

## RESULTS

Pathology materials of 421 of 438 (92%) cases were independently reviewed. Of those re-reviewed, 11 (3%) were deemed non-cancerous by the study pathologist. In addition to these cases, we excluded the tumours from a subject who had multiple tumours of two histologies and tumours from the 15 non-white subjects. Of the remaining tumours, we performed mutational analysis on 330 tumours, and assessed persistent staining and staining intensity in 356 tumours. There were no significant clinical or demographic differences comparing those patients included and excluded for p53 study. The overall prevalence of p53 mutation was 9.1% (30 out of 330; Table 1

**Table 1:** Prevalence of p53 mutation, p53 IHC persistent staining (⩾50%) and p53 IHC strong intensity (3+)

	**p53 mutation**		**p53 persistent staining**		**p53 strong intensity**		
	***N* (prevalence)**	**Prevalence OR (95% CI)^a^**	***N* (prevalence)**	**Prevalence OR (95% CI)^a^**	***N* (prevalence)**	**Prevalence OR (95% CI)^a^**	**Totals^b^**
All subjects	30 (9.1)		236 (66.3)		89 (25.0)		356 (100.00)
							
*Gender*							
Female	10 (13.3)	1.0 (reference)	58 (69.9)	1.0 (reference)	18 (21.7)	1.0 (reference)	83
Male	20 (7.8)	0.6 (0.3–1.2)	178 (65.2)	0.9 (0.7–1.3)	71 (26.0)	0.9 (0.5–1.5)	273
							
*Age*							
<55	8 (12.3)	1.0 (reference)	47 (67.1)	1.0 (reference)	17 (24.3)	1.0 (reference)	70
55–65	4 (3.7)	0.3 (0.1–1.1)	81 (68.1)	1.0 (0.7–1.5)	31 (26.1)	1.0 (0.6–1.8)	119
>65	18 (11.5)	0.9 (0.4–2.1)	108 (64.7)	1.0 (0.7–1.4)	41 (24.6)	0.8 (0.4–1.4)	167
							
*Histology*							
Transitional cell	29 (8.9)	1.0 (reference)	233 (66.4)	1.0 (reference)	85 (24.2)	1.0 (reference)	351
Nontransitionalcell	1 (20.0)	0.7 (0.1–5.8)	3 (60.0)	1.2 (0.4–3.7)	4 (80.0)	0.9 (0.3–2.5)	5
							
*Stage*							
Noninvasive, G1–G2	9 (4.6)	1.0 (reference)	150 (68.8)	1.0 (reference)	13 (6.0)	1.0 (reference)	218
Noninvasive, G3	6 (19.4)	4.4 (1.5–12.5)	15 (46.9)	0.7 (0.4–1.2)	8 (25.0)	4.5 (1.8–10.9)	32
*In situ*	1 (10.0)	2.5 (0.3–21.2)	8 (80.0)	1.2 (0.6–2.5)	8 (80.0)	14.5 (5.8–35.9)	10
Invasive	14 (15.4)	3.4 (1.4–8.0)	63 (65.6)	1.0 (0.7–1.3)	60 (62.5)	10.8 (5.9–19.9)	96
							
*Disease group*^c^							
TaG1-2	9 (4.6)	1.0 (reference)	150 (68.8)	1.0 (reference)	13 (6.0)	1.0 (reference)	218
CIS	1 (10.0)	2.6 (0.3–21.4)	8 (80.0)	1.2 (0.6–2.5)	8 (80.0)	14.7 (5.9–36.4)	10
TaG3/T1	12 (14.3)	3.2 (1.3–7.6)	49 (55.1)	0.8 (0.6–1.1)	40 (44.9)	8.0 (4.2–15.0)	89
T2	8 (21.1)	4.8 (1.8, 12.8)	29 (74.4)	1.1 (0.7–1.7)	28 (71.8)	12.4 (6.3–24.2)	39

a Prevalence ORs adjusted for all other variables in the table except disease group.

b Mutation data available on 330 subjects.

c Prevalence ORs adjusted for gender, age and histology.

). There was no statistically significant difference in mutation prevalence by gender or age, although there were more mutations in women than men. The prevalence of p53 mutation increased from 4.6% for the noninvasive low-grade tumours to 19.4% for the noninvasive high-grade tumours (POR=4.4; 95% CI=1.5–12.5) and to 15.4% for invasive tumours (POR=3.4; 95% CI=1.4–8.0). Likewise, the prevalence of mutation clearly increased with more advanced disease group (using the Larsson classification (Larsson *et al*, 2003)), with a POR of 4.8 (95% CI=1.8–12.8) for T2 tumours compared to TaG1-G2 tumours (Table 1).

The same tumours were evaluated for p53 presence using immunohistochemical staining, using separate criteria for the extent of staining (⩾50% of the cells in the tumour having evidence of persistent p53 protein) and intensity (scored as 0, 1, 2 or 3). When these data were analysed, we also observed no effect of gender or age on inactivation of p53 (Table 1). Strong intensity of staining (3 *vs* <3) was associated with tumour invasiveness and grade; 63% (60 out of 96) of the invasive tumours had an intensity rating of 3, while only 6% (13 out of 218) of the noninvasive tumours were intensely stained (POR=10.8; 95% CI=5.9–19.9). Again, with the Larsson disease group classification (Larsson *et al*, 2003), the POR for T2 tumours was increased for intense p53 staining (POR=12.4; 95% CI=6.3–24.2) compared to TaG1–G2 tumours. There was a much less marked association of positive IHC staining with invasive disease when the criteria were based on the proportion of positively staining cells (⩾50%) (Table 1).

Table 2

**Table 2:** Concordance between: (a) p53 mutation and p53 IHC staining; (b) p53 IHC persistent staining and p53 IHC strong intensity

	**(a) p53 Mutation**						
	**(neg) *N* (%)**	**(pos) *N* (%)**	**Total *N***	**Specificity**	**Sensitivity**	**(Pos) (95% CI) predictive value**	**(Neg) (95% CI) predictive value**
*IHC persistent* (>*50*%)				0.34 (0.29–0.39)	0.77 (0.72–0.81)	0.10 (0.07–0.14)	0.94 (0.91–0.96)
+ (pos)	197 (65.9)	23 (76.7)	220				
− (neg)	102 (34.1)	7 (23.3)	109				
							
*IHC intensity* (*3*+)				0.77 (0.72–0.82)	0.53 (0.48–0.59)	0.19 (0.15–0.23)	0.94 (0.92–0.97)
+ (pos)	69 (23.1)	16 (53.3)	85				
− (neg)	230 (76.9)	14 (46.7)	244				
	299 (100.0)	236 (100.0)	329 (100.0)				
							
	**(b) IHC persistent (⩾50%)**						
	**(neg) *N* (%)**	**(pos) *N* (%)**	**Total *N***	**Specificity**	**Sensitivity**	**(Pos) (95% CI) predictive value**	**(Neg) (95% CI) predictive value**
*IHC intensity* (*3*+)				0.83 (0.80–0.87)	0.29 (0.25–0.34)	0.78 (0.73–0.82)	0.37 (0.32–0.42)
+ (pos)	20 (16.7)	69 (29.2)	89				
− (neg)	100 (83.3)	167 (70.8)	267				
	120 (100.0)	236 (100.0)	356 (100.0)				

(a) and (b) provides the relationship between the different measures of p53 alteration. The sensitivity and specificity were both higher for intense IHC staining than persistent staining when compared with p53 mutation (Table 2 (a)). Persistent staining alone was far more prevalent than intense staining, although there was a significant correlation between the two measures (Table 2 (b)).

In an effort to refine our estimates of the clinical factors associated with alterations of the p53 pathway, we compared the tumours containing both a p53 mutation and evidence of inactivation by IHC with those that were wild type for IHC and mutation (Table 3

**Table 3:** Concordant p53 mutation and p53 IHC staining by demographic and clinical characteristics

	**p53 mutation/persistent staining**			**p53 mutation/intensity**		
	**p53 wt/IHC <50%**	**p53 mutant/IHC>50%**	**OR (95% CI)^a^**	**p53 wt/IHC <3**	**p53 mutant/IHC 3+**	**OR (95% CI)^a^**
**Characteristic**	***N* (%)**	***N* (%)**		***N* (%)**	***N* (%)**	
*Sex*						
Female	22 (21.6)	9 (39.1)	1.0 (reference)	54 (23.5)	6 (37.5)	1.0 (reference)
Male	80 (78.4)	14 (60.9)	0.5 (0.2–1.6)	176 (76.5)	10 (62.5)	0.4 (0.1–1.4)
						
*Age (year)*						
<55	18 (17.6)	7 (30.4)	1.0 (reference)	44 (19.1)	4 (25.0)	1.0 (reference)
55–65	34 (33.3)	3 (13.0)	0.2 (0.0–0.9)	78 (33.9)	3 (18.8)	0.4 (0.1–2.2)
>65	50 (49.0)	13 (56.5)	0.4 (0.1–1.5)	108 (47.0)	9 (56.2)	0.5 (0.1–2.2)
						
*Stage*						
Noninvasive	71 (70.3)	10 (43.4)	1.0 (reference)	197 (86.0)	4 (25.0)	1.0 (reference)
Invasive	30 (29.7)	13 (56.5)	1.5 (0.5–4.7)	32 (14.0)	12 (75.0)	3.3 (0.8–13.0)
						
*Grade*						
G1	25 (24.8)	2 (8.7)	1.0 (reference)	109 (47.6)	0	—
G2	41 (40.6)	4 (17.4)	1.4 (0.2–8.8)	82 (35.8)	2 (12.5)	1.0 (reference)
G3	35 (34.7)	17 (73.9)	6.3 (1.0–38.6)	38 (16.6)	14 (87.5)	9.0 (1.7–47.5)
						
*Disease group*^b^						
TaG1-2	57 (55.9)	6 (26.1)	1.0 (reference)	178 (77.4)	2 (12.5)	1.0 (reference)
CIS	1 (1.0)	0	—	1 (0.4)	0	—
TaG3/T1	36 (35.3)	10 (43.5)	3.1 (0.9–9.9)	42 (18.3)	7 (43.7)	17.6 (3.3–92.9)
T2	8 (7.8)	7 (30.4)	10.6 (2.5–45.5)	9 (3.9)	7 (43.7)	77.4 (13.0–460.1)

a ORs are adjusted for all other variables in the table except disease group.

b ORs are adjusted for gender and age.

). P53 inactivation clearly increased with tumour grade (Table 3). Also, the classification of bladder cancers by disease group indicated a substantial increase in prevalence of both persistent and intense p53 staining in the presence of mutation associated with more aggressive disease (Table 3).

We further examined whether the pattern of p53 staining was related to a part of the p53 gene (i.e. a specific exon). As is evident in Table 4

**Table 4:** p53 mutation by exon, tumour stage and immunohistochemical result

**p53 mutation**		p53 Immunohistochemical staining*	**Tumour stage**
**Exon**	**Total *N***	***N* (%) Intense (3+)**	***N* (%) invasive**
5	7	3 (43%)	2 (29)
6	3	1 (33)	2 (67)
7	10	5 (50)	5 (50)
8	5	5 (100)	4 (80)
9	5	2 (40)	1 (25)

* *P*=0.02 for exon 8 *vs* others.

, all of the mutations in exon 8 were associated with intense staining. Four of the five tumours with exon 8 mutations were invasive. Compared with all other exons, there was a significant association of intense staining with a mutation in exon 8 of the p53 gene (*P*=0.02).

## DISCUSSION

We used a population-based approach to examine somatic inactivation of the p53 gene in bladder cancer. We observed the overall prevalence of p53 mutation to be approximately 10%. This is similar to that reported by Berggren *et al* (2001) in a large, non-hospital-based study in Sweden. Other studies that selected for hospitalised cases and specifically patients who undergo cystectomy report much higher frequencies of p53 mutation (Sidransky *et al*, 1991; Fujimoto *et al*, 1992; Cordon-Cardo *et al*, 1994). This is likely attributable to the clear association of p53 mutation with disease grade and stage. In our study (as well as almost all others), the prevalence of mutation increases with degree of invasiveness, and hospitalised patients will certainly be more likely to have more invasive disease that requires medical intervention. This suggests that p53 mutations normally occur late in the evolution of bladder tumours and that they are associated with a rapid, invasive phenotype.

The significance of the discordance between IHC-positive tumours and p53 mutation is unclear, although it is commonly reported (Gao *et al*, 2000; Babjuk *et al*, 2002; Doak *et al*, 2003). This discordance may suggest several hypotheses. It is often assumed that stabilisation of nuclear protein indicates that the protein is mutant. However, the presence of wild-type protein may indicate that p53 is functionally inactive because of alterations in the pathways that lead to its activation. Candidates for alteration in this pathway clearly include the MDM2 protein, which is responsible for catalysing the ubiquitination and degradation of p53. MDM2 is often altered in bladder cancer (Lu *et al*, 2002), and thus should be evaluated in concert with examination of p53 for persistent and intense staining, as well as with mutation. Regardless of the mechanism, in the case where the p53 protein is normal, but persistent staining is detectable, its function is most likely dramatically altered. It is clearly possible (perhaps even likely) that tumour clones with mutations in the p53 gene have features distinct from tumours that have other alterations in the pathway.

Importantly, our data suggest that the histologic character of staining (i.e. intensity of staining *vs* percent) is more indicative of tumour aggressiveness. Intense staining might occur when p53 is completely inactivated and the protein becomes more densely distributed in the nucleus of the cell. At the same time, an increased number of cells with positive (but less intense) staining may reflect a distinct form of pathway inactivation where activation or the protein is altered. Our data are consistent with this hypothesis in that we found at least some specificity for mutations; all of the mutations in exon 8 were associated with intense staining.

Finally, our data further suggest that additional work is needed to fully understand the underlying reason for the discordance between p53 protein persistence in early-stage bladder cancer. It seems clear that multiple mechanisms of p53 alteration are detected by immunohistochemical staining, and these differences may be associated with the characteristics of the bladder tumour. In addition, detection of aberrant p53 protein (along with proteins in the p53 pathway) using antibody-mediated methods can likely be made more sensitive and specific for detecting clinically important endpoints.
